# GJD Modulates Cardiac/Vascular Inflammation and Decreases Blood Pressure in Hypertensive Rats

**DOI:** 10.1155/2022/7345116

**Published:** 2022-09-17

**Authors:** Shadi A. D. Mohammed, Hanxing Liu, Salem Baldi, Pingping Chen, Fang Lu, Shumin Liu

**Affiliations:** ^1^Graduate School of Heilongjiang University of Chinese Medicine, Harbin, 150040 Heilongjiang, China; ^2^Research Center of Molecular Diagnostics and Sequencing, Axbio Biotechnology (Shenzhen) co., Ltd., Gaoxin South 7th Road, Yuehai Street, Nanshan District, Shenzhen, China; ^3^Institute of Traditional Chinese Medicine, Heilongjiang University of Chinese Medicine, Harbin, 150040 Heilongjiang, China

## Abstract

Gedan Jiangya decoction (GJD) (aqueous ethanol extract), a traditional Chinese medicine formula which contain six botanical drugs (Uncaria rhynchophylla (Miq.) Miq., Salvia miltiorrhiza Bunge, Pueraria lobata (Willd.) Ohwi, Eucommia ulmoides Oliv., Prunella vulgaris L., and Achyranthes bidentata Blume) was designed to treat hypertension; however, the underlying mechanism of action is unclear. This study aimed to determine the mechanisms of action of GJD in the treatment of hypertension in spontaneously hypertensive rats (SHR). Male SHRs were randomly divided into five groups: GJD doses were low (1.36 g/kg/d), medium (2.72 g/kg/d), and high (5.44 g/kg/d), captopril (13.5 mg/kg/d), and SHR groups, with Wistar-Kyoto rats (WKY) serving as the control. Every rat was gavaged once a day. The ALC-NIBP, a noninvasive blood pressure device, measured systolic (SBP) and diastolic (DBP) blood pressures. Six weeks following treatment, all rats were anesthetized. The blood samples were obtained from the abdominal aorta and then serum isolated to assess endothelin-1 and angiotensin II, interleukin-1beta, interleukin-6, and TNF-alpha. The left ventricular and thoracic aortas were taken for HE staining, immunohistochemistry, RT-qPCR, and western blot examination. Following GJD therapy, SBP and DBP were significantly lowered, as were serum levels of endothelin-1 and angiotensin II. The thickness of the left ventricular and thoracic aorta walls reduced, as did type I collagen, type III collagen, and alpha-SMA expression in the left ventricular and aortic tissues. The GJD treatment significantly reduced serum levels of the inflammatory markers interleukin-1beta, interleukin-6, and TNF-alpha. Furthermore, interleukin-1 beta, interleukin-6, TNF-alpha, TAK1, and NF-*κ*B/p65 levels were significantly reduced in left ventricular and aortic tissues, whereas IkB-alpha levels were significantly elevated. GJD has a dose-dependent effect on all parameters. In conclusion, GJD has been shown to lower blood pressure, improve cardiovascular remodeling, and reduce inflammation via regulating NF-*κ*B in SHRs.

## 1. Introduction

Hypertension is the leading cause of cardiovascular disease-related morbidity and mortality worldwide [1]. Globally, the prevalence of SBP levels more than 110 mm Hg has increased over the past three decades; in 2015, the number of fatalities and disability-adjusted life years (DALYs) was 10 million and 212 million, respectively, representing a 1.4-fold increase since 1990 [1]. One million persons from 44 low and middle income countries were studied; 17.5% were hypertensive; 39.2% were diagnosed with high blood pressure; and 29.9% were treated, yet only 10.3% had their hypertension under control [2]. Uncontrolled hypertension is associated with a higher risk of cardiovascular disease death [3, 4]. Furthermore, people with hypertension have a higher chance of developing cardiovascular disease (CVD) throughout their lives, and the onset of CVD morbidity occurs five years earlier in those with hypertension compared to those with normal blood pressure [5, 6]. Over 2.54 million people in China died in 2017 due to hypertension-related disorders, with about 69% of those deaths attributable to stroke, 54% to ischemic heart disease, and 41% to other forms of cardiovascular disease, according to a study by the Chinese Center for Disease Control and Prevention (CCDC) [7].

Despite the availability of effective hypertension drug therapy, there has been a rise in hypertension-related cardiovascular mortality, as seen by current US statistics, in the decreased percentage of persons with controlled hypertension from 53.8% in 2013 to 2014 to 43.7% from 2017 to 2018 [8]. Furthermore, among Chinese individuals with hypertension, 46.9% were aware of their disease, 40.7% were taking prescribed western antihypertensive medication, and only 15.3% had their hypertension under control [9]. This might be partly due to the numerous medications available not effectively targeting the necessary pathways, as well as counter-regulatory mechanisms generated by these therapies limiting their blood pressure (BP-)-lowering effect. As a result, new therapeutic methods are desperately required. Thus, traditional Chinese medicine can provide different treatment methods according to the cause and the response of the body, and multicomponent, multitarget, and multipathway pharmacological action properties have shown consistent therapeutic advantages on chronic difficult disorders, attracting increasing worldwide interest [10]; [11, 12]. Finding effective drugs for treating hypertension from traditional Chinese medicine has become a major technique in developing innovative antihypertension medications.

Cardiovascular remodeling and inflammation are essential in hypertensive target organ damage's etiology and adaptive mechanism [13, 14]. Several studies have shown that the activation of the nuclear factor-B (NF-*κ*B) pathway results in an upregulation of inflammatory factors such as interleukin-1 beta, interleukin-6, and tumor necrosis factor-alpha [15, 16]. Hypertension may be treated with TCM that have anti-inflammatory effects and the potential to stop hypertensive cardiovascular remodeling. This study investigates the possible mechanism of GJD, for which a Chinese patent publication number (No.: CN114246896A) was previously applied, but the underlying mechanism has not yet been entirely detailed. GJD includes six botanical drugs, namely, Uncaria rhynchophylla (Miq.) Miq. [Rubiaceae] (Gouteng), Salvia miltiorrhiza Bunge [Lamiaceae] (Danshen), *Pueraria lobata* (Willd.) Ohwi [Fabaceae] (Gegen), Eucommia ulmoides Oliv. [Eucommiaceae] (Duzhong), Prunella vulgaris L. [Lamiaceae] (Xiakucao), and Achyranthes bidentata Blume [Amaranthaceae] (Niuxi). Uncaria rhynchophylla is a component of the Chinese crude medication Gouteng, which is extensively used to cure various conditions such as anti-inflammatory, decreased hypertension, and cardiac fibrosis [17]; [18]. Pharmacological effects on the cardiovascular system from Salvia miltiorrhiza, Pueraria lobata, and Eucommia ulmoides include anti-inflammatory, endothelium protecting, antioxidative, vasodilatory, and myocardial protective effects [19, 20]; [21]. Modern pharmacological investigations have demonstrated that Prunella vulgaris and Achyranthes bidentata offer cardiovascular therapeutic effects such as blood pressure reduction, anti-inflammatory, and antioxidant properties [22, 23].

Due to the major role of inflammation in hypertension and cardiovascular remodeling, we propose that GJD reduces hypertension-related inflammation and might be utilized as an antihypertensive medication to minimize cardiovascular remodeling. This study aimed to observe whether GJD may be utilized as a new antihypertensive medicine by evaluating its effects on heart and aorta remodeling and inflammation via NF-*κ*B regulation in SHR.

## 2. Materials and Methods

### 2.1. Plant Material and Extraction

The following GJD botanical drugs included in the study: Uncaria rhynchophylla (Miq.) Miq. (Lot No. 20200901), 10 g; Salvia miltiorrhiza Bunge, (Lot No. 20191001) 25 g; Pueraria lobata (Willd.) Ohwi (Lot No. 20191001) 30 g; Eucommia ulmoides Oliv. (Lot No. 20190901), 15 g; Prunella vulgaris L. (Lot No. 20190701) 15 g; and Achyranthes bidentata Blume (Lot No. 20200901) 20 g were purchased from Heilongjiang Xiushengtang Pharmaceutical Co., Ltd.

Salvia miltiorrhiza, Pueraria lobata, Eucommia ulmoides, Prunella vulgaris, and Achyranthes bidentata were mixed with a 60% aqueous ethanol solution at a material-liquid ratio of 1 g dissolved in 10 ml, soaked for 30 minutes, heated and refluxed the soaking solution for 1.5 hours (keeping the solution slightly boiling during the reflux process), and then filtered through six layers of degreasing gauze. The components were then filtered after being subjected to a 1.5-hour period of reheating and refluxing with 10 times their original volume of 60% aqueous ethanol solution. After that, we combined the two filtrates, distilled the ethanol under reduced pressure using a rotary evaporator, and dried the solution under reduced pressure and vacuum to get extracts powder of Salvia miltiorrhiza, Pueraria lobata, Eucommia ulmoides, Prunella vulgaris, and Achyranthes bidentata. Similarly, Uncaria rhynchophylla was soaked in a 70% aqueous ethanol solution in a 1 g: 10 ml ratio for 0.5 hour, heated to 65~ 75°C, and soaked again for 2 hours. Uncaria rhynchophylla was also filtered through six layers of degreasing gauze and soaked in 10 times the amount of 70% aqueous ethanol solution for 2 hours. The two filtrates were then combined, distilled the ethanol under reduced pressure using a rotary evaporator, and then dried under reduced pressure and vacuumed to obtain extracts powder of Uncaria rhynchophylla. Finally, two extracts were mixed to obtain GJD powder.

### 2.2. Animal

Under the experimental animal approval license number SYXK (Hei)2018-007, a total of 35 SHR and 7 Wistar Kyoto (WKY) control male rats (Vital River Laboratory Animal Technology Co., Ltd., Beijing), 10 weeks of age (body weight 250 ± 10 g), were utilized in the investigation. Throughout the experiment, all rats had unrestricted access to normal chow and tap water ad libitum, and they were kept in a room with a regulated temperature (21 ± 3°C), humidity (50 ± 6%), and lighting (12 h/12 h light-dark cycle). The study protocol was authorized by the Heilongjiang University of Chinese Medicine's Animal Care and Use Committee (Approval number: 2020031203).

### 2.3. Groups, Drug Doses, and Blood Pressure Measurement

After one week of acclimatization period, the 35 SHR were randomly assigned into the following 5 groups, each containing 7 rats: model group (SHR), the low dose of GJD group (GJD-LD) gavage of GJD (1.36 g/kg/d), the medium dose of GJD group (GJD-MD) gavage of GJD (2.72 g/kg/d), the high dose of GJD group (GJD-HD) gavage of GJD (5.44 g/kg/d), and captopril group (CAP): gavage of captopril (13.5 mg/kg/d Meilun Biotechnology Co., Ltd., Dalian). The GJD doses were dissolved in 1 ml of distilled water (every rat was gavaged 1 ml once time a day), and the control group (WKY) and model group (SHR) were gavaged with an equivalent volume (1 ml) of distilled water every day (at 8 a.m. feeding the rats and at 9 a.m. gastric gavage).

Body weight, systolic, and diastolic blood pressure (SBP and DBP) were measured every week. The systolic and diastolic blood pressures were measured with the tail-cuff method using ALC-NIBP, a noninvasive blood pressure system from Shanghai Alcott Biotechnology Co., Ltd., China. BP measurement was performed first, then gavaging, and the measurements were taken three times per rat, and an average value was reported. After 6 weeks, the rats were euthanized with sodium pentobarbital (45 mg/kg, intraperitoneally), and then, the blood samples were taken from the abdominal artery and immediately centrifuged at 1176 g for 15 minutes at 4°C and stored at -80°C. The hearts were retrieved and cleaned in normal saline before being dried using filter paper. The atrium's free walls, major blood arteries, and right ventricle were removed along the atrioventricular junction, and the hearts' left ventricles were obtained. The left ventricular mass index (LVMI) was determined to measure thickening of the left ventricular as follows: LVMI = left ventricular weight (mg)/body weight (g). The left ventricular and thoracic aorta parts were frozen and kept at -80°C for subsequent western blot and RT-qPCR analyses. In addition, sliced sections of the left ventricular and thoracic aorta were fixed in 4% paraformaldehyde for HE staining and immunohistochemistry analysis.

### 2.4. ELISA Measurement of Endothelin-1, Angiotensin II, and Inflammatory Factors in Serum

The ELISA kits were used to assess serum levels of interleukin-1 beta (Nanjing Jiancheng Bioengineering Institute, Nanjing, China, Serial number: H002), interleukin-6 (Nanjing Jiancheng Bioengineering Institute, Nanjing, China, Serial number: H007-1-2), TNF-alpha (Nanjing Jiancheng Bioengineering Institute, Nanjing, China, Serial number: H052-1), endothelin-1 (Nanjing Jiancheng Bioengineering Institute, Nanjing, China, Serial number: H093), and angiotensin II (Nanjing Jiancheng Bioengineering Institute, Nanjing, China, Serial number: H185). All kit procedures were completed in line with the company's instructions.

### 2.5. Western Blot

The total protein was obtained from the thoracic aorta and left ventricular of three rats selected randomly using RIPA lysis buffer with PMSF and phosphatase inhibitor (Servicebio, China). Protein concentration was measured using a BCA protein detection kit (Servicebio, China). SDS-PAGE separated the whole protein, which was subsequently transferred to 0.45 *μ*M on the PVDF membrane (Servicebio, China). The membrane was sealed with 5% nonfat milk for 1 hour at room temperature before being incubated with the primary antibody overnight at 4°C. The membrane was then washed three times with TBST, incubated for one hour at room temperature with a secondary antibody, and the protein was identified using an ECL reagent (Servicebio, China). Primary antibodies include TAK1 (GB11564, 1: 1,000, Servicebio, China), IKB-alpha (GB13212-1, 1 : 1,000, Servicebio, China), p65 (GB11997, 1 : 1,000, Servicebio, China), and *β*-actin (GB15001, 1: 2,000, Servicebio, China).

### 2.6. HE Staining

Fresh tissues were taken from thoracic aorta and left ventricle, fixed in 4% paraformaldehyde at 4°C for 24 hours, and then dehydrated. After drying and embedding in paraffin, the fixed tissue was cut into 5-micron thick slices and stained with hematoxylin and eosin. Using an Eclipse Ci-L (Nikon, Japan) optical microscope to select the target area of the tissue for 20× imaging, try to fill the whole field of vision with the tissue. At least three 200× visual fields were randomly selected from each slice and observed under a microscope. HE-stained slides were evaluated by two veterinary pathologists to identify abnormalities.

### 2.7. Immunohistochemistry

The tissue sections were deparaffinized with xylene and then dehydrated using a graded alcohol series (put the sections into BioDewax and clear solution I for 15 minutes–BioDewax and clear solution II for 15 minutes–BioDewax and clear solution III for 15 minutes–absolute ethanol I for 5 minutes–absolute ethanol II for 5 minutes–85% alcohol for 5 minutes–75% alcohol for 5 minutes–rinsed in distilled water). To block endogenous peroxidase activity, the sections were incubated in 3% H₂O₂ for 25 minutes at room temperature in the dark and then washed with phosphate-buffered saline (PBS). Sections were blocked for 30 minutes at room temperature before being incubated overnight at 4°C with diluted primary antibodies (interleukin-1 beta, interleukin-6, TNF-alpha, type I collagen, type III collagen, and alpha-SMA). After washing in phosphate buffered saline, the sections were incubated for 50 minutes with the matching secondary antibodies. Then, 3,3-diaminobenzidine (DAB, Servicebio, China) was used as a chromogen, and slices were counterstained with hematoxylin. Dehydration and mounting are accomplished by soaking the section in a series of solvents until it is dehydrated and transparent, as follows: 75% alcohol for 5 minutes, 85% alcohol for 5 minutes, absolute ethanol I for 5 minutes, anhydrous ethanol II for 5 minutes, n-butanol for 5 minutes, and xylene I for 5 minutes. SweSuper clean BioMount medium was then used to mount the sections. Primary antibodies used for detection were against interleukin-1 beta (bs-0812R, 1 : 100, Bioss, China), interleukin-6 (GB11117, 1 : 200, Servicebio, China), TNF-alpha (GB11188, 1 : 200, Servicebio, China), type I collagen (GB11022-3, 1 : 800, Servicebio, China), type III collagen (GB111629, 1 : 250, Servicebio, China), and alpha-SMA (GB111364, 1 : 300, Servicebio, China). All the above antibodies are rabbit polyclonal antibodies. The Eclipse Ci-L light microscope was used to randomly select the target area of the tissue for 200 × imaging. Image pro plus 6.0 analysis software is used to measure the integrated optical density (IOD) values in three visual field slices of each slice with the pixel area as the standard unit.

### 2.8. Quantitative Real-Time Polymerase Chain Reaction (RT-qPCR)

The total RNA was extracted from the left ventricular and thoracic aorta tissues of three rats selected at random using Trizol reagent (Takara), reversed transcription into cDNA using the primescripttm RT Kit (Takara), and then subjected to real-time PCR reaction using TB Green® Premix Ex Taq™ II (Tli RNaseH Plus) (Takara), and the gene expression level was quantified using QuantStudio™3 real-time PCR system. After a hot start (42°C 2 min and 4°C 5 min) (37°C 15 min, 85°C 5 s, and 4°C 5 min), amplification was performed (stage 1 Reps 1 (95.0°C and 0.30 min), stage 2 Reps 40 (95.0°C, 0.05 min, 60.0°C, 0.34 min), and stage 3 Reps 1 (95.0°C, 0.15 min, 60.0°C 1.00 min, 95.0°C, 0.15 min). The data was calculated and analyzed using the 2^-*ΔΔ*Ct^ technique, and GAPDH acted as an internal control.  Table 1 shows the primers used for PCR amplification.

### 2.9. Statistical Analysis

In this study, all data are presented as mean ± standard deviation (SD), and they were statistically analyzed using Graphpad Prism 7. Statistical differences were assessed using one-way ANOVA with Tukey's multiple comparisons as a post hoc test. Statistical significance was defined as *P* < 0.05.

## 3. Results

### 3.1. The Effect of GJD on Blood Pressure

**Figure 1:**
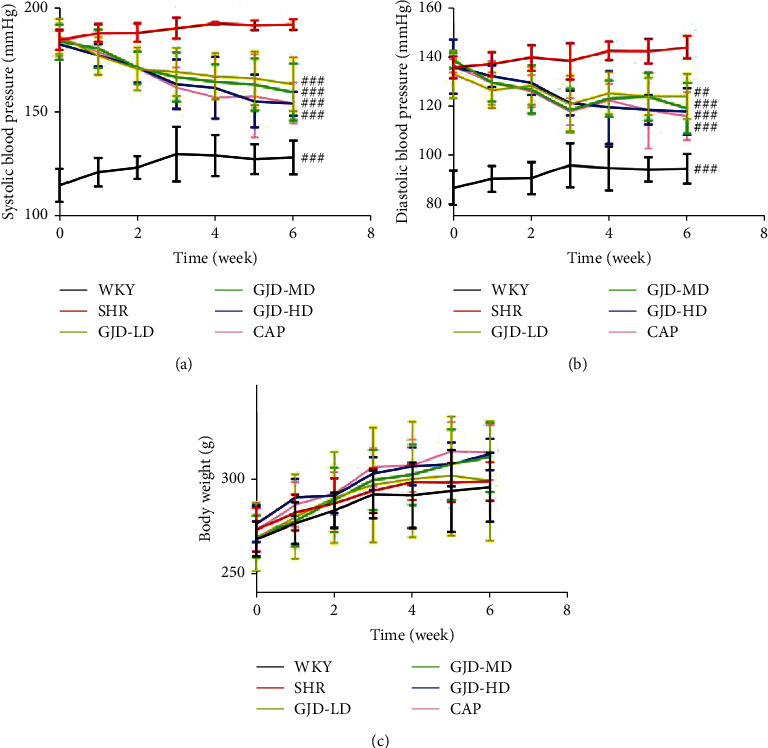
Effect of GJD on systolic and diastolic blood pressures and body weight. (a) SBP, (b) DBP, and (c) body weight. WKY indicates the WKY control group, SHR indicates the SHR Model group, GJD-LD indicates the SHR treated with GJD at a low dose, GJD-MD indicates the SHR treated with GJD at a medium dose, GJD-HD indicates the SHR treated with GJD at a high dose, and CAP indicates SHR treated with captopril. Data are means ± SD, *n* = 7. ^#^*P* < 0.05, ^##^*P* < 0.01, ^###^*P* < 0.001 vs. SHR group.

After 6 weeks of GJD gavage therapy, both systolic and diastolic blood pressures are significantly reduced, as seen in Figures 1(a) and 1(b). The systolic blood pressure was in the WKY (128.34 mm Hg), SHR (191.79 mm Hg), GJD-LD (163.45 mm Hg), GJD-MD (159.40 mm Hg), GJD-HD (153.84 mm Hg), and CAP group (154.26 mm Hg), while the diastolic blood pressure in the WKY (94.44 mm Hg), SHR (143.86 mm Hg), GJD-LD (124.20 mm Hg), GJD-MD (119.18 mm Hg), GJD-HD (117.86 mm Hg), and CAP group (116.13 mm Hg). The rats in GJD-HD experienced the highest decrease in systolic and diastolic blood pressures (Figures 1(a) and 1(b)), but there were no substantial differences between the GJD-HD and CAP groups. We also observed no differences in body weight across groups (Figure 1(c)).

### 3.2. Effects of GJD on Endothelin-1 and Angiotensin II

**Figure 2:**
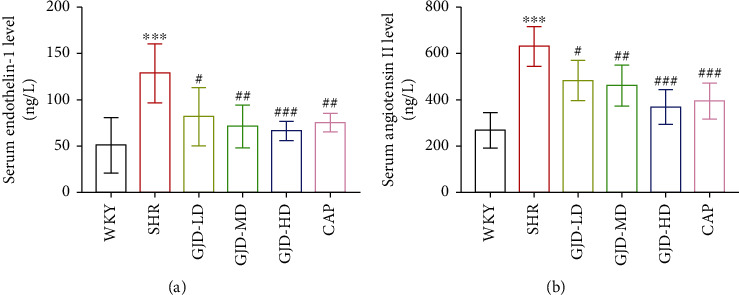
Serum level of endothelin-1 and angiotensin II. WKY indicates the WKY control group, SHR indicates the SHR model group, GJD-LD indicates the SHR treated with GJD at a low dose, GJD-MD indicates the SHR treated with GJD at a medium dose, GJD-HD indicates the SHR treated with GJD at a high dose, and CAP indicates SHR treated with captopril. Data are means ± SD, *n* = 7. ^∗^*P* < 0.05, ^∗∗^*P* < 0.01, ^∗∗∗^*P* < 0.001 vs. WKY group, ^#^*P* < 0.05, ^##^*P* < 0.01, ^###^*P* < 0.001 vs. SHR group.

After 6 weeks of gavage treatment, we measured the serum's endothelin-1 and angiotensin II levels. The level of endothelin-1 and angiotensin II in the GJD-LD, GJD-MD, GJD-HD, and CAP groups was considerably lower than in the model group (Figures 2(a) and 2(b)). Both endothelin-1 and angiotensin II levels were decreased significantly in the GJD-HD group (Figures 2(a) and 2(b)).

### 3.3. GJD's Effect on Nuclear Factor Kappa B

**Figure 3:**
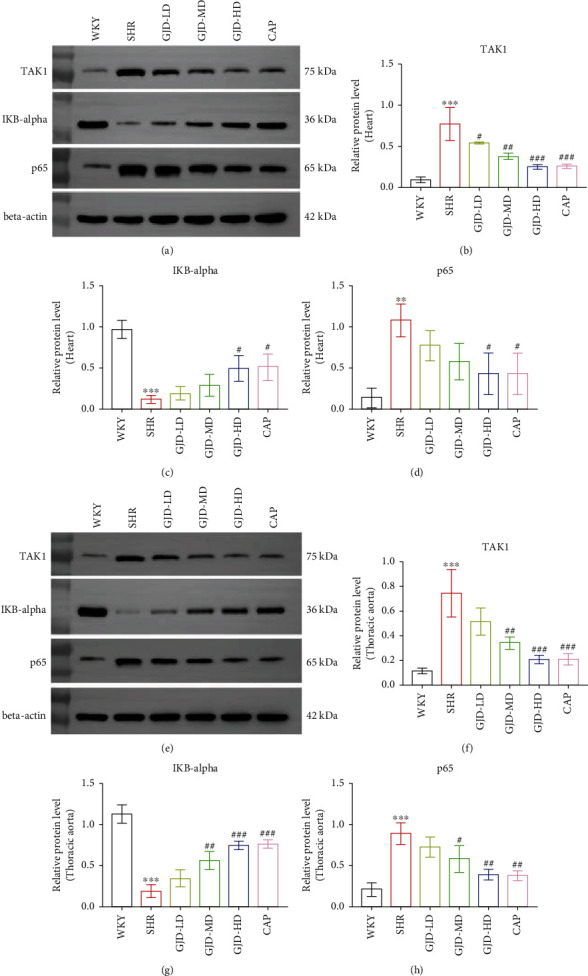
Effect of GJD on the expression of TAK1, IkB-alpha, and p65. The expression of TAK1, IkB-alpha, and p65 in the left ventricular and aorta was examined by western blot. (a) The left ventricular expression of TAK1, IkB-alpha, and p65, and (b) the aorta expression of TAK1, IkB-alpha, and p65. The bar charts indicate relative protein levels referenced to *β*-actin. WKY indicates the WKY control group, SHR indicates the SHR model group, GJD-LD indicates the SHR treated with GJD at a low dose, GJD-MD indicates the SHR treated with GJD at a medium dose, GJD-HD indicates the SHR treated with GJD at a high dose, and CAP indicates SHR treated with captopril. Data are means ± SD. *n* = 3. ^∗^*P* < 0.05, ^∗∗^*P* < 0.01, ^∗∗∗^*P* < 0.001 vs. WKY group. ^#^*P* < 0.05, ^##^*P* < 0.01, ^###^*P* < 0.001 vs. SHR group.

After six weeks of therapy, we measured TAK1, p65, and IkB-alpha proteins in the left ventricular and thoracic aorta. TAK1 and P65 levels in SHR increased significantly in comparison to the WKY group; however, IkB-alpha levels declined significantly. In the left ventricular, TAK1 decreased significantly in the GJD-LD, GJD-MD, GJD-HD, and CAP groups compared to the SHR group, while p65 decreased significantly in the GJD-HD and CAP groups in comparison to the SHR group, but the lowered level of p65 in GJD-LD and GJD-MD was not significant. While IkB-alpha increased significantly in the GJD-HD and CAP groups, it did not increase significantly in the GJD-LD and GJD-MD groups (Figure 3(a)).

The expression of p65 and TAK1 in the thoracic aorta of SHRs in the GJD-MD, GJD-HD, and CAP groups was considerably lower than in the SHR group, however, the decrease in GJD-LD was not statistically significant, Conversely, the level of IkB-alpha in the aorta of SHRs in the GJD-MD, GJD-HD, and CAP groups was significantly higher than in the SHR group; however the increased in GJD-LD was not significant (Figure 3(b)). The rats in the GJD-HD group had the highest elevation in IkB-alpha levels, as well as the lowest levels of TAK1 and p65 (Figure 3(b)).

**Figure 4:**
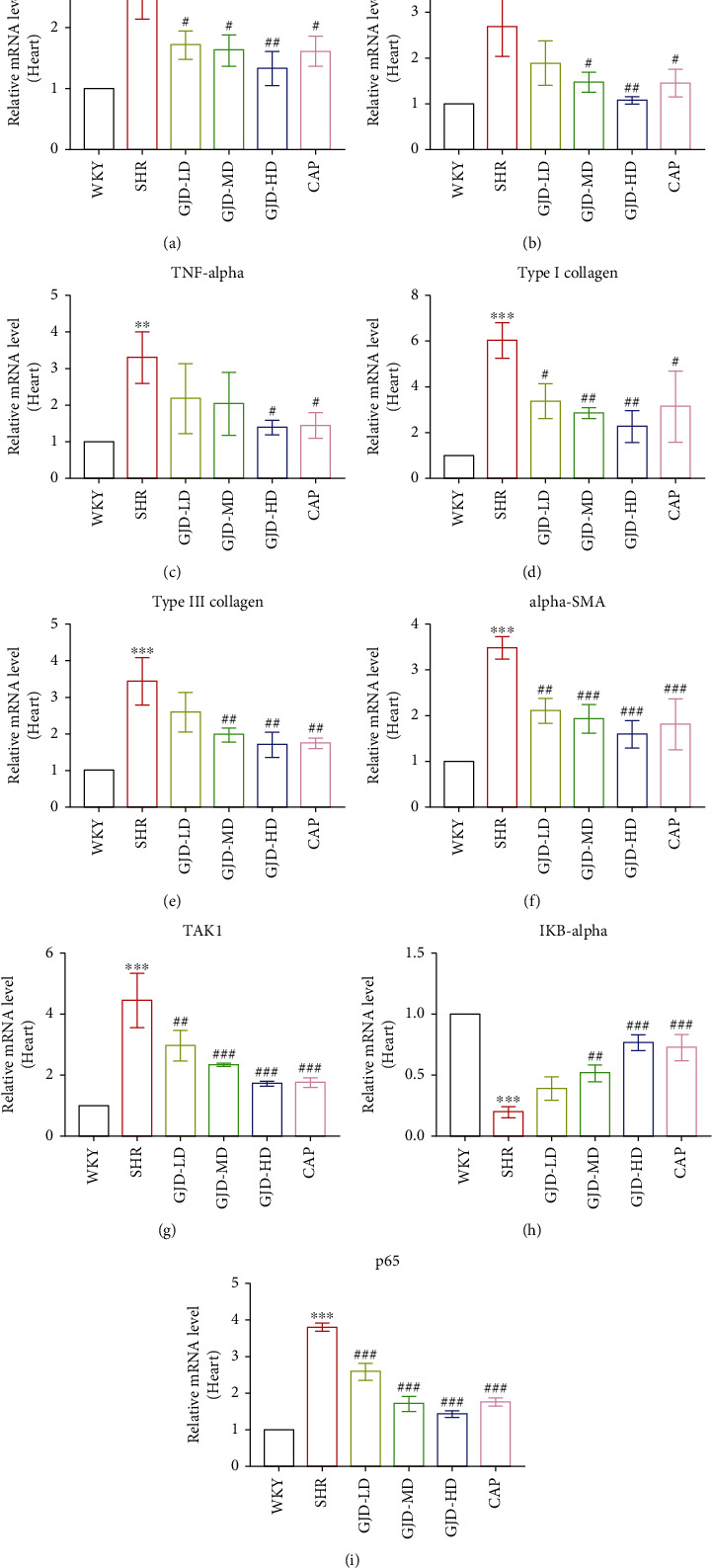
Effects of GJD on mRNA expression of interleukin -1 beta, interleukin-6, TNF-alpha, type I collagen, type III collagen, and alpha-SMA in the left ventricular. (a) Interleukin-1 beta, (b) interleukin-6, (c) TNF-alpha, (d) type I collagen, (e) type III collagen, (f) alpha-SMA, (g) TAK1, (h) IkB-alpha, and (i) p65. WKY indicates the WKY control group, SHR indicates the SHR model group, GJD-LD indicates the SHR treated with GJD at a low dose, GJD-MD indicates the SHR treated with GJD at a medium dose, GJD-HD indicates the SHR treated with GJD at a high dose, and CAP indicates SHR treated with captopril. Data are means ± SD, *n* = 3. ^∗^*P* < 0.05, ^∗∗^*P* < 0.01, ^∗∗∗^*P* < 0.001 vs. WKY group, ^#^*P* < 0.05, ^##^*P* < 0.01, ^###^*P* < 0.001 vs. SHR group.

**Figure 5:**
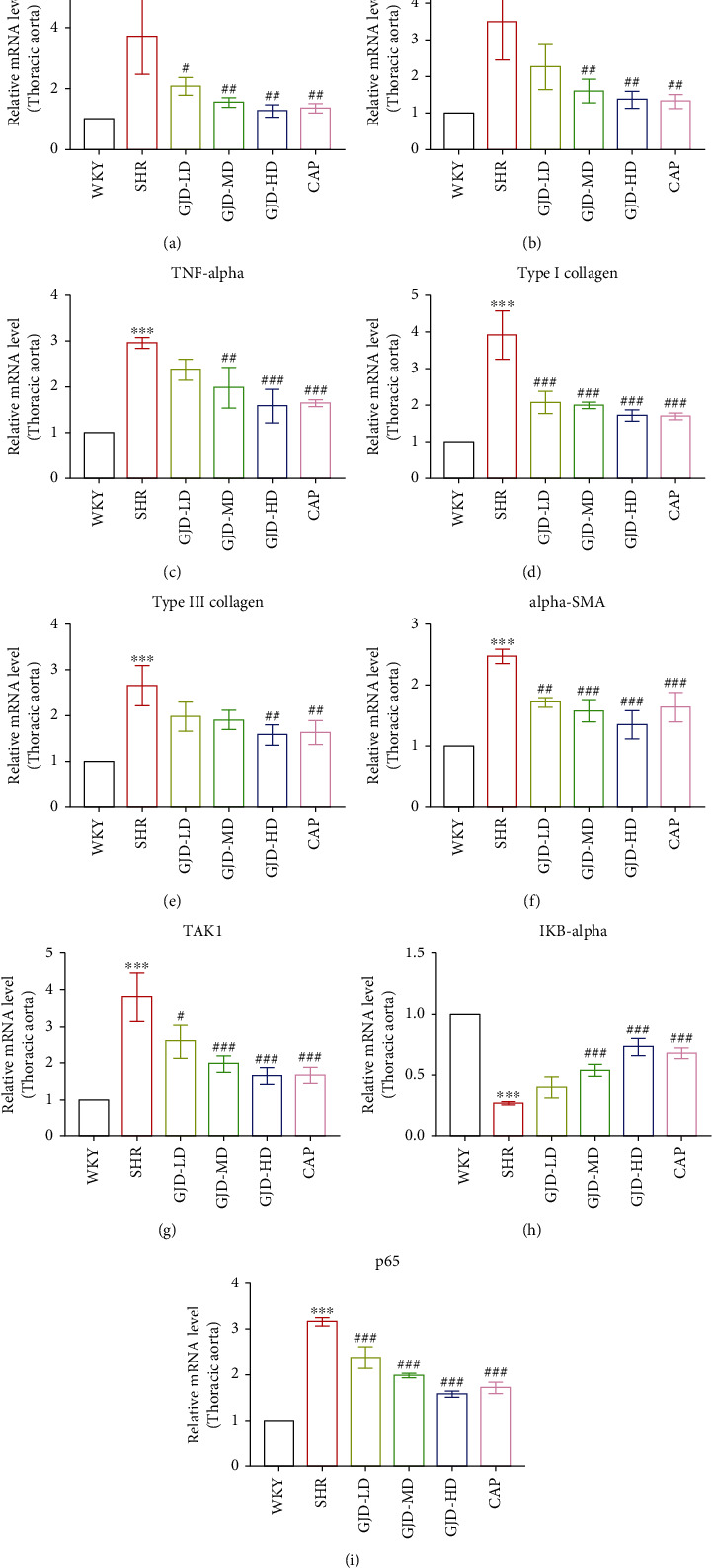
Effects of GJD on mRNA expression of interleukin-1 beta, interleukin-6, TNF-alpha, type I collagen, type III collagen, and alpha-SMA in the thoracic aorta. (a) Interleukin-1 beta, (b) interleukin-6, (c) TNF-alpha, (d) type I collagen, (e) type III collagen, (f) alpha-SMA, (g) TAK1, (h) IkB-alpha, and (i) p65. WKY indicates the WKY control group, SHR indicates the SHR model group, GJD-LD indicates the SHR treated with GJD at a low dose, GJD-MD indicates the SHR treated with GJD at a medium dose, GJD-HD indicates the SHR treated with GJD at a high dose, and CAP indicates SHR treated with captopril. Data are means ± SD. *n* = 3. ^∗^*P* < 0.05, ^∗∗^*P* < 0.01, ^∗∗∗^*P* < 0.001 vs. WKY group, ^#^*P* < 0.05, ^##^*P* < 0.01, ^###^*P* < 0.001 vs. SHR group.

The Real-time PCR also examined TAK1, IkB-alpha, and p65 mRNA expression in the left ventricular and thoracic aorta. As shown in Figures 4 and 5(g)–5(i), the mRNA expressions of p65 and TAK1 were decreased significantly in the left ventricular and thoracic aorta of SHRs in the GJD-LD, GJD-MD, GJD-HD, and CAP groups in comparison to those in the SHR group, whereas the level of IkB-alpha was significantly higher in both the left ventricular and aorta of SHRs in the GJD-MD, GJD-HD and CAP groups compared to those in SHR group, however, the decrease in GJD-LD was not significant.

### 3.4. GJD's Effect on Inflammatory Cytokines

**Figure 6:**
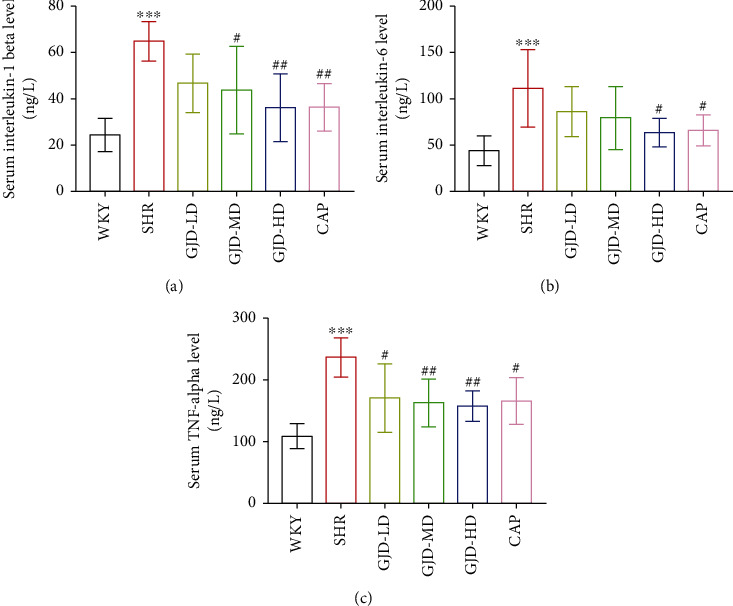
Serum level of interleukin-1 beta, interleukin-6, and TNF-alpha in each group. (a) endothelin-1, (b) angiotensin II, (c) interleukin-1 beta, (d) interleukin-6, and (e) TNF-alpha. WKY indicates the WKY control group, SHR indicates the SHR model group, GJD-LD indicates the SHR treated with GJD at a low dose, GJD-MD indicates the SHR treated with GJD at a medium dose, GJD-HD indicates the SHR treated with GJD at a high dose, and CAP indicates SHR treated with captopril. Data are means ± SD, *n* = 7. ^∗^*P* < 0.05, ^∗∗^*P* < 0.01, ^∗∗∗^*P* < 0.001 vs. WKY group, ^#^*P* < 0.05, ^##^*P* < 0.01, ^###^*P* < 0.001 vs. SHR group.

After six weeks of gavage therapy, the levels of the markers of inflammation interleukin-1 beta, interleukin-6, and TNF-alpha were significantly reduced in the SHR's serum in the GJD-LD, GJD-MD, GJD-HD, and CAP groups in comparison to the SHR group (Figures 6(a)–6(c)). The rats in the GJD-HD group exhibited a significant decrease in the markers of inflammation interleukin-1 beta, interleukin-6, and TNF-alpha (Figures 6(a)–6(c)), whereas there were no significant differences between the GJD-HD and the CAP groups.

**Figure 7:**
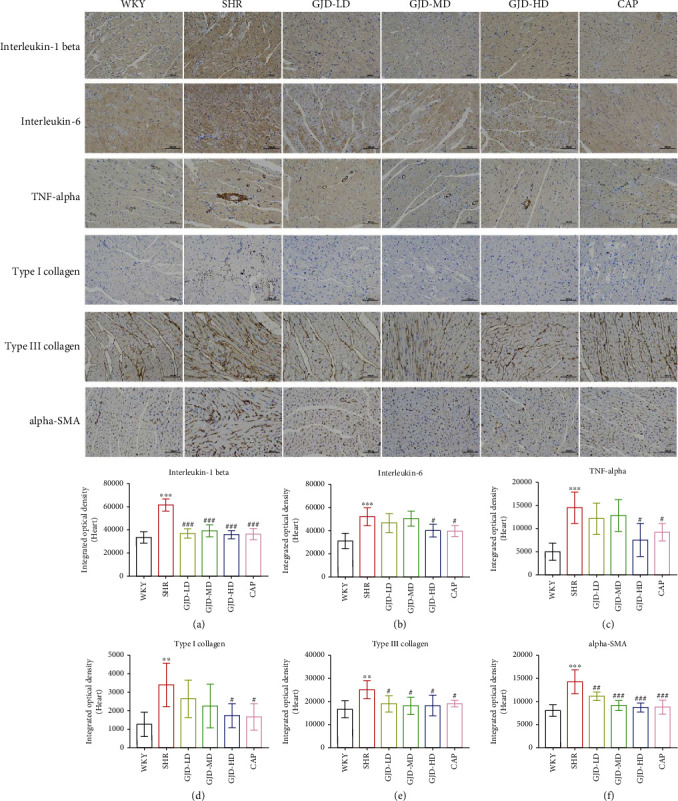
In the left ventricular, immunohistochemistry sections and quantification of protein expressions of interleukin-1 beta, interleukin-6, TNF-alpha, type I collagen, type III collagen, and alpha-SMA. (A) Interleukin-1 beta, (B) interleukin-6, (C) TNF-alpha, (D) type I collagen, (E) type III collagen, and (F) alpha-SMA. The images were taken at a magnification of ×200. Scale bar = 100 *μ*m. WKY indicates the WKY control group, SHR indicates the SHR model group, GJD-LD indicates the SHR treated with GJD at a low dose, GJD-MD indicates the SHR treated with GJD at a medium dose, GJD-HD indicates the SHR treated with GJD at a high dose, and CAP indicates SHR treated with captopril. Data are means ± SD. *n* = 7. ^∗^*P* < 0.05, ^∗∗^*P* < 0.01, ^∗∗∗^*P* < 0.001 vs. WKY group, ^#^*P* < 0.05, ^##^*P* < 0.01, ^###^*P* < 0.001 vs. SHR group.

**Figure 8:**
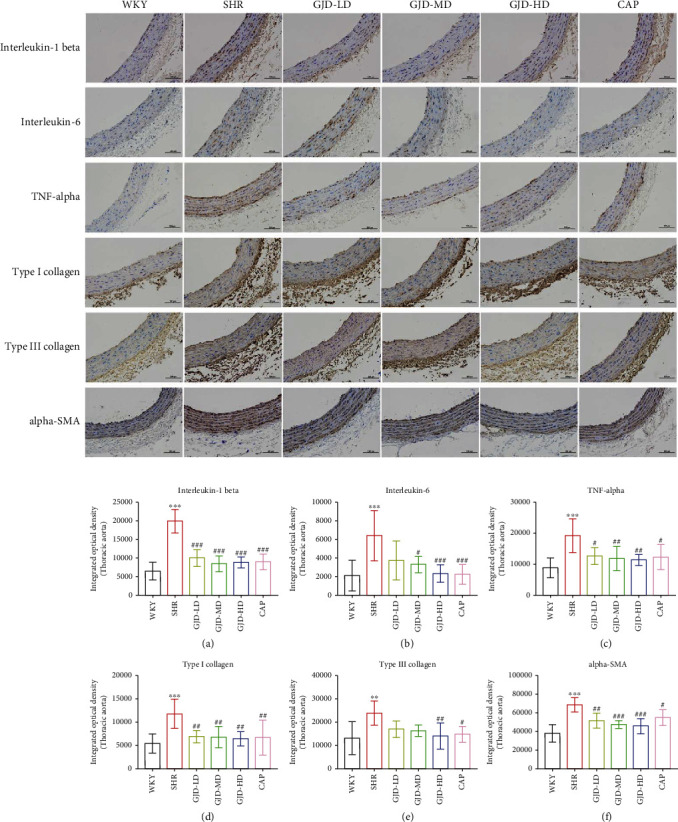
In the thoracic aorta, immunohistochemistry sections and quantification of protein expressions of interleukin -1 beta, interleukin-6, TNF-alpha, type I collagen, type III collagen, and alpha-SMA. (A) Interleukin-1 beta, (B) interleukin-6, (C) TNF-alpha, (D) type I collagen, (E) type III collagen, and (F) alpha-SMA. The images were taken at a magnification of ×200. Scale bar = 100 *μ*m. WKY indicates the WKY control group, SHR indicates the SHR model group, GJD-LD indicates the SHR treated with GJD at a low dose, GJD-MD indicates the SHR treated with GJD at a medium dose, GJD-HD indicates the SHR treated with GJD at a high dose, and CAP indicates SHR treated with captopril. Data are means ± SD. *n* = 7. ^∗^*P* < 0.05, ^∗∗^*P* < 0.01, ^∗∗∗^*P* < 0.001 vs. WKY group, ^#^*P* < 0.05, ^##^*P* < 0.01, ^###^*P* < 0.001 vs. SHR group.

Furthermore, we assessed the expression of interleukin-1 beta, interleukin-6, and TNF-alpha in the left ventricular and aorta by immunohistochemistry. In the left ventricle, the interleukin-1 beta, interleukin-6, and TNF-alpha expression in the model group (SHR) were markedly higher than WKY group. In comparison to the SHR group, all GJD groups had significantly lower interleukin-1 beta expression, but the low and medium dosages have no significant decrease in interleukin-6 and TNF-alpha expression in the left ventricle (Figures 7(a)–7(c)). The rats in the GJD-HD group had the greatest decrease in markers of inflammation interleukin-1 beta, interleukin-6, and TNF-alpha, while there were no differences between the GJD-HD group and the CAP group (Figures 7(a)–7(c)). In addition, in the aorta, the interleukin-1 beta, interleukin-6, and TNF-alpha expression in the SHR group were significantly higher than WKY group (Figures 8(a)–8(c)). In comparison with the SHR group, GJD-MD and GJD-HD groups had significantly lower interleukin-1 beta, interleukin-6, and TNF-alpha expression; however, GJD-LD only decreased interleukin-1 beta and TNF-alpha expression markedly, but the interleukin-6 did not decrease significantly in this group.

The real-time PCR was also performed to examine interleukin-1 beta, interleukin-6, and TNF-alpha mRNA expression in the left ventricular and aorta. As shown in Figures 4(a)–4(c), interleukin-1 beta, interleukin-6, and TNF-alpha are decreased in the left ventricular of SHRs in the GJD-LD, GJD-MD, and GJD-HD in comparison to those in the SHR group (IL-1 beta decreased significantly in all treatment group; however, the GJD-MD, and GJD-HD significantly reduced the interleukin-6, but the TNF-alpha only significantly decreased in GJD-HD). The rats in the GJD-HD group had the greatest significant decrease in interleukin-1 beta, interleukin-6, and TNF-alpha in the left ventricular (Figures 4(a)–4(c)). In the aorta, interleukin-1 beta, interleukin-6, and TNF-alpha expression reduced in all GJD groups (interleukin-1 beta decreased significantly in all treatment groups; however, interleukin-6 and TNF-alpha reduced significantly in both GJD-MD and GJD-HD) (Figures 5(a)–5(c)). The rats in the GJD-HD group had the greatest significantly decreased mRNA expression of interleukin-1 beta, interleukin-6, and TNF-alpha in the thoracic aorta, while no significant differences were identified between the GJD-HD group and CAP group (Figures 5(a)–5(c)).

### 3.5. GJD's Effects on Cardiovascular Remodeling

**Figure 9:**
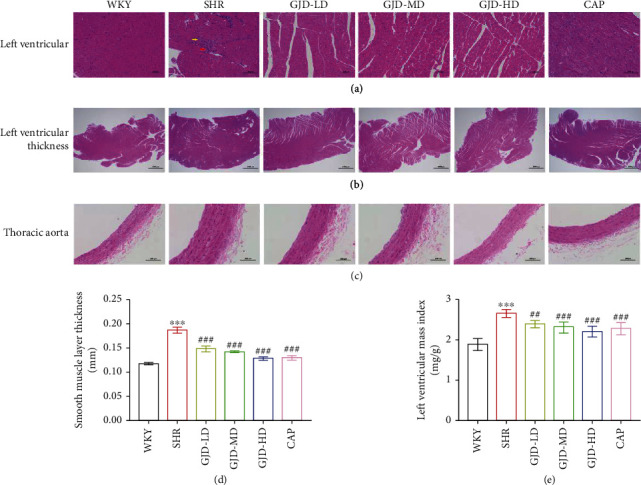
Effect of GJD on cardiovascular remodeling and histology of left ventricular and thoracic aorta of SHR after 6-week treatment by GJD. (a) HE staining Left ventricular (magnification of ×200, scale bar 100 *μ*m). (b) Ventricular wall (magnification of ×20, scale bar 1000 *μ*m). (c) HE staining of thoracic aorta (magnification of ×200, scale bar 100 *μ*m). (d) Smooth muscle layer thickness. (e) LVMI. Partial myocardial fibrosis necrolysis was replaced by hyperplastic connective tissue (yellow arrows) with punctate lymphocytic infiltration (red arrows) in the SHR group. WKY indicates the WKY control group, SHR indicates the SHR model group, GJD-LD indicates the SHR treated with GJD at a low dose, GJD-MD indicates the SHR treated with GJD at a medium dose, GJD-HD indicates the SHR treated with GJD at a high dose, and CAP indicates SHR treated with captopril. Data are means ± SD, *n* = 7. ^∗^*P* < 0.05, ^∗∗^*P* < 0.01, ^∗∗∗^*P* < 0.001 vs. WKY group. ^#^*P* < 0.05, ^##^*P* < 0.01, ^###^*P* < 0.001 vs. SHR group.

Based on the findings of left ventricular and thoracic aorta HE staining following six weeks of gavage administration, when compared with the WKY group, the heart tissue from SHRs showed significant thickening of the ventricular wall by an increase in LVMI, partial myocardial fiber necrosis, accompanied by fibrosis and mild inflammation. It was observed that after therapy, LVMI are reduced to a greater extent in the GJD-LD, GJD-MD, GJD-HD, and CAP groups than in the SHR group, and there is no necrosis or fibrosis in these groups as shown in Figures 9(a), 9(b), and 9(e). Neither the GJD-LD nor the GJD-MD groups showed any signs of necrosis or fibrosis, and the distinction between the two groups was unclear. Groups GJD-HD and CAP both showed no signs of necrosis or fibrosis, and the difference between them was not apparent (Figure 9(a)).

**Figure 10:**
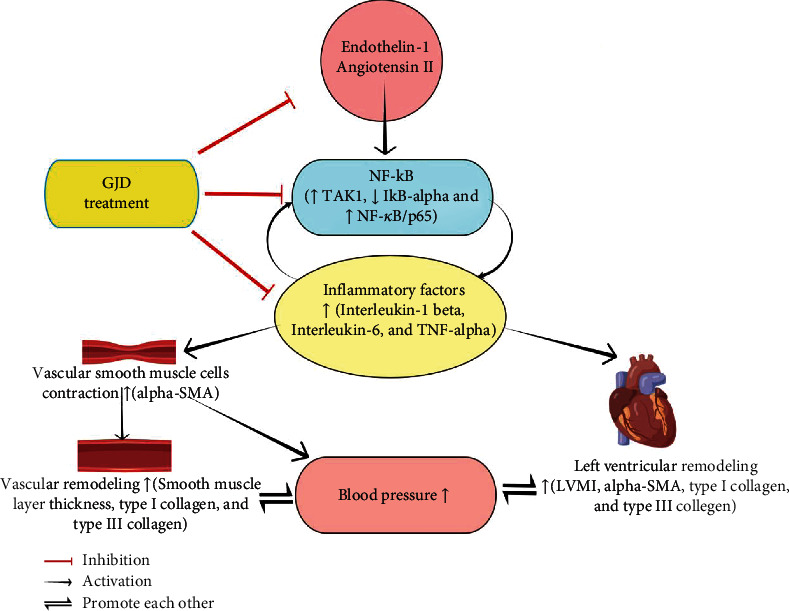
Flowchart of possible mechanisms of GJD attenuates hypertension and cardiovascular remodeling through inhibition NF-*κ*B, created by FigDraw.

As shown in Figures 9(c) and 9(d), we measure the thoracic aorta thickness, and it is lower in the GJD-LD, GJD-MD, GJD-HD, and CAP groups than in the SHR group. GJD and CAP groups significantly suppressed the development of aortic hypertrophy compared to the SHR group. The rats in the GJD-HD group had the highest decrease in thoracic aorta wall thickness (Figures 9(c) and 9(d)). Additionally, the immunohistochemistry and RT-qPCR analysis indicated that the expression of type I collagen, type III collagen, and alpha-SMA in the left ventricular and thoracic aorta was significantly decreased in GJD's groups than SHR group, but there were no differences between CAP and GJD-HD groups (Figures 4(d)–4(f), 5(d)–5(f), 6(d)–6(f), 7(d)–7(f) and 8(d)–8(f)). The possible mechanisms of GJD attenuate hypertension and cardiovascular remodeling (Figure 10).

## 4. Discussion

In this research, we investigated the efficacy of GJD in treating hypertension. SHRs were treated with various dosages of GJD, and the efficacies were assessed on numerous parameters. This research will provide new concepts and theoretical foundations for managing hypertensive related target organ remodeling. In this research, we examined how blood pressure changed following GJD therapy. We observed that the following six weeks of GJD gavage therapy at GJD-LD, GJD-MD, and GJD-HD doses significantly reduced SBP and DBP. We observed that GJD-HD had the largest decrease in SBP and DBP after six weeks of treatment. Angiotensin II and endothelin-1 are the main vasoconstrictors in hypertension pathogenesis [24–26]. Moreover, numerous studies found that increased Ang II and ET-1 activated NF-*κ*B signaling pathway [27–30]. Here, after therapy with GJD groups, the serum's levels of endothelin-1and angiotensin II were significantly reduced, with GJD-HD having the most significant reduction.

Several studies have implicated the nuclear factor-kappa B (NF-B) pathway in the pathogenesis of hypertensive cardiovascular disease through its role in the transcriptional activation of inflammatory markers [15, 31, 32]; [33, 34]. Signal-induced degradation of IkB proteins initiates NF-*κ*B activation [35, 36], which happens primarily through phosphorylation and activation of a kinase termed IkB kinase (IKK). TAK1 causes degradation of IkB via the IkB kinase (IKK) and nuclear factor (NF) kappa B (NF-B) signaling pathways [37]; [38, 39]. With the degradation of IkB, the NF-*κ*B complex is free to enter the nucleus and trigger the expression of certain genes that have NF-*κ*B DNA-binding sites nearby [40–42]. Rodríguez-Iturbe B et al. showed that long-term suppression of this proinflammatory transcription factor could prevent hypertension in this model, confirming the early increase in NF-*κ*B activation in the SHR [43].

The inflammatory markers interleukin-1 beta, interleukin-6, and tumor necrosis factor alpha were all secreted at higher rates after NF-kappaB activation [44–46]. Recent studies have shown that interleukin-1beta, interleukin-6, and TNF- alpha are all elevated in high blood pressure, suggesting that these inflammatory markers may play a role in the pathogenesis of hypertension [7, 47–49]; [50]. Numerous studies have shown that nuclear factor (NF)-kappaB (NF-*κ*B) signaling is crucial in inflammation and cardiovascular diseases [33, 34, 51, 52]. Many major CVD, including hypertension, are caused by abnormal vascular smooth muscle cells (VSMC) contraction, migration, and proliferation [53–55]. The primary component of the artery's medial layer is VSMC [54]. These cells contract to control blood vessel tone (constriction/dilation), hence controlling blood flow and pressure [56, 57]. VSMC may also secrete molecules, which allows for the creation and repair of extracellular matrix proteins as well as the modulation of vascular wall structure [58]. Strong expression of several marker genes, such as alpha-SMA, SM22, and calponin, is characteristic of contractile VSMC, which are nonproliferative and fully differentiated [59, 60]. In brief, VSMC contraction is caused by alpha-SMA, the major and particular isoform of actin produced in VSMC [54, 55]. In mice, a deficiency of alpha-SMA can result in reduced VSMC contractibility and then hypotension [61, 62]. Hence, the ability of VSMC to differentiate and contract depends on the expression level of gene that serves as marker for these cells. Numerous studies have shown that inflammatory indicators, which are activated by NF-KB, cause vascular smooth muscle cells (VSMC) to become more contractile and, thus, are responsible for elevations in blood pressure. In the Choi S study, it shows that the TNF*α*-induced VSMC phenotypic alteration and vasodilatory dysfunction were blocked by NF-*κ*B inhibition [63]. Data suggested that IL-1B treatment elevated proinflammatory genes through an NF-*κ*B-dependent pathway, and initial observations suggested a connection between inflammation/IL-1B and its associated proteins and alterations in the phenotypic of smooth muscle cells in systemic arterial hypertension [64, 65]. In this research, the same is found that NF-*κ*B induced the inflammatory markers interleukin-1 beta, interleukin-6, and TNF-alpha which upregulates the alpha-SMA expression and that a sign of VSMC contraction in SHR group may lead to increase blood pressure, while the downregulation of alpha-SMA expression levels in GJD groups suggests that GJD has the capacity to prevent VSMC differentiation from the proliferative type. As a result, GJD may be functionally relevant for lowering blood pressure by decreasing the contractile phenotype of VSMCs.

Furthermore, the proinflammatory cytokines have been linked to pathological cardiac hypertrophy [66]. Many studies over the last two decades have shown that IL-1*β*, IL-6, and TNF-*α* are intimately linked to cardiac fibrosis, pathological cardiac remodeling, and cardiac hypertrophy [67–70], and the inflammatory cytokines induced following the activation of the nuclear factor-*κ*B (NF-*κ*B) pathway [71] [44]. Therefore, inhibition of the NF-*κ*B pathway might be a means to decrease inflammation led to attenuate cardiac remodeling. In the study of Miguel-Carrasco JL, it is demonstrated that captopril reduces inflammation in the left ventricle of hypertensive rats and suggests that NF-*κ*B-driven inflammatory reactivity may be responsible for this impact by inactivating of NF-B-dependent proinflammatory factors [34]. Our study also showed that GJD decreased hypertrophy of the left ventricle by inhibiting NF-*κ*B-related inflammatory markers.

Increase in wall thickness of the aorta and the thickening of the ventricular wall are hallmark features of hypertension-induced widespread cardiovascular remodeling [72–74]. Vascular remodeling in hypertension comprises alterations to smooth muscle cells in the artery wall, as well as endothelial cells, elastin, and collagen levels [75]. These vascular remodeling characteristics in hypertension are typically associated with a renin-angiotensin-aldosterone system imbalance, endothelial cell (EC) dysfunction, contractile properties, and phenotypic switching of VSMC, as well as extracellular matrix (ECM) reorganization and inflammation throughout the entire vessel wall [53]. In addition, myofibroblasts are differentiated from cardiac fibroblasts by myocardial injury, and the expression of alpha-SMA signals effective transformation into a phenotype with a high capacity to synthesize extracellular matrix proteins [76, 77], and the fibrillary proteins which are the primary components of the cardiac extracellular matrix are type I collagen and type III collagen, which are considered as indicators of fibrosis in fibroblasts and then remodeling [76, 78]. For that type I collagen, type III collagen and alpha-SMA expression promote thoracic and left ventricular remodeling [79–82]. The Salvia miltiorrhiza active ingredients have the effect of reducing the alpha-SMA expression, collagen, and ET-1, thereby reducing the vessel wall thickness [83]. Icariside II may reduce the formation of alpha-SMA and type I collagen/type III collagen in SHRs via the MMP/TIMP-1 and TGF-b1/Smad2,3/p-p38 signaling pathways, according to the findings of Fu's study [84]. Here, GJD therapy decreased arterial wall thickness and ventricular wall thickening in SHRs, and type I collagen, type III collagen, and alpha-SMA levels in the thoracic aorta and left ventricle were significantly reduced following GJD therapy.

NF-*κ*B signaling activation may enhance the expression of inflammatory markers in the SHR thoracic aorta and left ventricle leading to hypertension and cardiovascular remodeling. GJD administration inhibited this change, suggesting that inflammatory component control through NF-*κ*B activation may be implicated in GJD's cardiovascular protective impact on SHR rats.

## 5. Conclusion

In conclusion, GJD may reduce blood pressure and improve left ventricular and aortic remodeling in a SHR model of hypertension by inhibiting inflammatory factors through NF-*κ*B. Our data indicated that GJD might be used as an antihypertensive agent and provides scientific basis for further pharmacological studies and clinical applications. This study has some limitations, such as the need to fully evaluate the active ingredients in the GJD and then extract the active ingredients to investigate further its impact on treating hypertension, and the GJD's regulatory mechanisms for improving hypertension symptoms in SHR models should be investigated further by assessing mRNA, ncRNA, and protein levels. In addition, we did not follow up on blood pressure measurements after withdrawal or with long-term treatment in this study; further researches are needed to assess the efficacy of GJD after withdrawal and long-term therapy.

## Figures and Tables

**Table 1:** Primer pairs list used in RT-qPCR.

Gene	Forward primer (5′–3′)	Reverse primer (5′–3′)
IL1B (interleukin-1 beta)	CCCTGAACTCAACTGTGAAATAGCA	CCCAAGTCAAGGGCTTGGAA
IL6 (interleukin-6)	TTGGGACTGATGTTGTTG	TGTGGGTGGTATCCTCTGT
TNF (TNF-alpha)	TCAGTTCCATGGCCCAGAC	GTTGTCTTTGAGATCCATGCCATT
COL1A1 (type I collagen)	CCTGCCGATGTCGCTATCC	TTGCCTTCGCCCCTGAG
COL3A1 (type III collagen)	AGATGCTGGTGCTGAGAAG	TGGAAAGAAGTCTGAGGAAGG
ACTA2 (alpha-SMA)	TTCGTGACTACTGCTGAGCG	CTGTCAGCAATGCCTGGGTA
MAP3K7 (TAK1)	AGCAGAAACGACAAGGCACT	CAGCGAGACAGTGGATTTGA
NFKBIA (IkB-alpha)	CCCTGGAAAATCTTCAGACG	ACAAGTCCACGTTCCTTTGG
RELA(p65)	GACCTGGAGCAAGCCATTAG	CACTGTCACCTGGAAGCAGA
GAPDH	TGCACCACCAACTGCTTAG	GATGCAGGGATGATGTTC

## Data Availability

All data generated or analyzed during this study have been deposited in the documents [Supplementary Data 1 and Supplementary Data 2]. Further enquiries can be directed to the corresponding author.

## Abbreviations

GJD: Gedan Jiangya decoction
SBP: Systolic blood pressure
DBP: Diastolic blood pressure
SHR: Spontaneously hypertensive rats
WKY: Wistar-Kyoto rats
P65: NF-*κ*B/p65
TAK1: Transforming growth factor b-activated kinase 1
VSMC: Vascular smooth muscle cells
TCM: Traditional Chinese medicine.

## Supplementary Materials

Supplementary Materials The supplementary materials which include all data used in this study in Supplementary Data 1 and western blot in Supplementary Data 2.
