# Supplementary material for: GJD Modulates Cardiac/Vascular Inflammation and Decreases Blood Pressure in Hypertensive Rats
Source: Mediators Inflamm. 2022 Sep 17;2022:7345116. doi: 10.1155/2022/7345116 (PMC9509256; doi:10.1155/2022/7345116)
Supplement: Supplementary Materials — The supplementary materials which include all data used in this study in Supplementary Data 1 and western blot in Supplementary Data 2. [file 7345116.f1.zip › Supplementary Data 1.pdf]

Baseline	WKY	SHR	GJD-LD	GJD-MD	GJD-HD	CAP
1	114	184	188	170	190	172
2	113	182	170	189	181	177
3	132	184	193	182	170	192
4	113	194	192	174	180	180
5	109	178	179	189	178	192
6	114	184	191	188	186	194
7	109	185	191	193	191	192
Mean	114.65	184.56	186.31	183.53	182.36	185.70

1 week	WKY	SHR	GJD-LD	GJD-MD	GJD-HD	CAP
1	111	181	167	179	173	192
2	121	185	178	164	175	178
3	119	189	162	190	171	170
4	130	193	181	175	185	193
5	125	193	185	178	183	162
6	115	186	179	186	178	178
7	127	188	186	190	174	178
Mean	121.18	187.73	176.94	180.26	176.93	178.68

2 weeks	WKY	SHR	GJD-LD	GJD-MD	GJD-HD	CAP
1	122	193	168	166	166	181
2	128	191	152	172	178	166
3	131	188	164	161	170	162
4	120	182	179	174	172	168
5	121	182	179	166	184	181
6	115	188	180	175	167	164
7	126	192	172	184	163	175
Mean	123.26	188.12	170.57	171.05	171.34	170.99

3 weeks	WKY	SHR	GJD-LD	GJD-MD	GJD-HD	CAP
1	143	192	177	165	145	145
2	147	179	173	158	178	161
3	117	192	174	173	165	160
4	122	193	172	151	159	161
5	139	190	179	187	161	178
6	125	193	163	163	178	163
7	115	193	146	170	157	164
Mean	129.79	190.28	169.22	166.57	163.34	161.70

4 weeks	WKY	SHR	GJD-LD	GJD-MD	GJD-HD	CAP
1	124	193	162	158	147	149
2	130	192	163	174	167	156
3	128	193	180	151	159	154
4	126	193	185	167	188	152
5	147	193	163	171	168	169
6	115	192	155	170	144	172
7	133	192	161	160	158	147
Mean	129.11	192.79	167.04	164.46	161.44	157.12

5 weeks	WKY	SHR	GJD-LD	GJD-MD	GJD-HD	CAP
1	121	189	159	152	160	180
2	126	188	187	160	161	149
3	137	193	172	178	154	159
4	136	193	165	151	149	182
5	118	193	161	155	130	126
6	127	193	146	164	163	163
7	126	193	172	182	169	144
Mean	127.24	191.67	166.17	163.15	155.15	157.44

6 weeks	WKY	SHR	GJD-LD	GJD-MD	GJD-HD	CAP
1	139	187	146	145	162	153
2	129	193	181	157	157	165
3	138	192	168	156	151	148
4	129	192	171	166	158	171
5	119	194	167	141	150	151
6	121	195	147	175	155	145
7	122	191	163	176	145	147
Mean	128.34	191.79	163.45	159.40	153.84	154.26

Baseline	WKY	SHR	GJD-LD	GJD-MD	GJD-HD	CAP
1	82	134	146	135	140	131
2	89	135	119	140	137	137
3	101	140	122	135	136	139
4	85	143	141	140	118	136
5	82	136	137	142	127	136
6	87	130	135	142	153	139
7	81	132	131	136	142	136
Mean	86.88	135.72	133.09	138.49	136.31	136.42

1 week	WKY	SHR	GJD-LD	GJD-MD	GJD-HD	CAP
1	83	134	120	131	133	143
2	91	131	134	120	128	126
3	90	138	115	141	135	119
4	99	133	129	131	139	133
5	91	143	125	123	134	126
6	85	136	127	124	127	121
7	94	144	135	137	129	138
Mean	90.35	136.99	126.52	129.69	132.09	129.38

2 weeks	WKY	SHR	GJD-LD	GJD-MD	GJD-HD	CAP
1	85	147	127	132	122	139
2	95	137	119	125	137	126
3	94	142	123	107	131	124
4	91	136	143	123	128	120
5	93	131	131	132	133	125
6	79	143	125	134	129	120
7	98	142	130	134	126	128
Mean	90.71	139.60	128.25	126.65	129.47	126.08

3 weeks	WKY	SHR	GJD-LD	GJD-MD	GJD-HD	CAP
1	107	141	126	115	115	111
2	106	130	112	113	128	117
3	84	127	126	127	124	110
4	93	143	137	106	118	121
5	100	136	126	130	116	124
6	96	144	117	114	125	120
7	86	147	102	124	123	122
Mean	96.00	138.31	120.97	118.48	121.11	117.76

4 weeks	WKY	SHR	GJD-LD	GJD-MD	GJD-HD	CAP
1	89	148	121	127	108	120
2	86	143	133	131	125	118
3	96	144	134	109	119	114
4	92	140	130	124	143	123
5	111	136	113	129	121	120
6	87	146	116	120	96	128
7	102	141	130	122	125	134
Mean	94.77	142.58	125.59	123.10	119.67	122.51

5 weeks	WKY	SHR	GJD-LD	GJD-MD	GJD-HD	CAP
1	87	138	123	115	121	135
2	95	147	132	114	127	118
3	102	137	129	139	115	124
4	98	142	121	116	115	137
5	92	149	115	129	109	92
6	91	147	115	122	120	114
7	94	137	133	132	123	109
Mean	94.20	142.36	124.01	123.84	118.74	118.47

6 weeks	WKY	SHR	GJD-LD	GJD-MD	GJD-HD	CAP
1	104	144	114	110	131	113
2	92	146	138	113	129	125
3	99	138	133	112	112	107
4	98	144	126	124	120	132
5	91	153	123	110	113	117
6	87	143	114	133	115	105
7	90	140	120	132	105	113
Mean	94.44	143.86	124.20	119.18	117.86	116.13

Baseline	WKY	SHR	GJD-LD	GJD-MD	GJD-HD	CAP
1	261.3	292.0	252.7	268.4	279.5	272.5
2	257.9	277.2	244.5	261.4	278.1	283.4
3	283.4	267.9	278.1	268.7	285.6	270.1
4	268.5	260.2	281.5	282.5	257.4	286.4
5	261.4	286.1	262.9	264.2	270.9	263.6
6	270.1	265.4	269.5	256.2	283.2	254.5
7	278.0	266.3	297.9	287.5	282.2	282.5
Mean	268.66	273.59	269.59	269.84	276.70	273.29

1 week	WKY	SHR	GJD-LD	GJD-MD	GJD-HD	CAP
1	264.6	280.5	251.9	279.6	294.2	288.7
2	268.0	288.9	249.9	268.7	296.3	298.3
3	292.6	280.6	287.6	278.5	296.1	281.5
4	281.9	270.2	300.5	293.2	271.2	295.8
5	267.3	299.4	283.2	270.4	286.5	280.4
6	288.6	276.5	280.3	258.6	289.3	265.6
7	276.0	282.5	308.4	297.3	300.1	296.7
Mean	277.00	282.66	280.26	278.04	290.53	286.71

2 weeks	WKY	SHR	GJD-LD	GJD-MD	GJD-HD	CAP
1	277.6	269.7	260.3	285.7	295.1	301.4
2	275.1	303.2	257.4	282.5	299.8	304.2
3	291.4	290.8	296.0	284.2	298.2	282.9
4	288.5	275.5	312.4	312.6	279.1	294.2
5	276.2	304.1	293.5	286.3	297.8	285.3
6	299.1	285.3	292.3	264.0	278.3	276.5
7	278.3	283.3	320.9	309.8	292.4	303.4
Mean	283.74	287.41	290.40	289.30	291.53	292.56

3 weeks	WKY	SHR	GJD-LD	GJD-MD	GJD-HD	CAP
1	294.9	281.9	255.2	300.6	307.3	311.3
2	276.3	307.6	264.3	299.4	309.8	320.9
3	293.5	297.8	298.1	291.6	309.7	300.3
4	300.5	281.8	322.7	314.4	291.2	301.4
5	288.0	310.6	302.6	297.5	311.7	298.6
6	312.6	291.3	294.9	272.6	294.6	294.5
7	279.1	287.5	342.1	321.2	296.9	317.6
Mean	292.13	294.07	297.13	299.61	303.03	306.37

4 weeks	WKY	SHR	GJD-LD	GJD-MD	GJD-HD	CAP
1	283.5	294.5	256.2	302.0	310.4	309.2
2	272.3	309.8	268.7	296.0	313.6	330.6
3	296.8	294.3	303.6	290.3	314.1	298.3
4	302.4	288.6	331.4	323.4	292.7	301.2
5	289.6	312.5	303.2	298.6	318.0	300.4
6	321.9	299.7	297.5	282.6	294.5	290.9
7	275.1	290.4	340.5	324.4	304.4	320.5
Mean	291.66	298.54	300.16	302.47	306.81	307.30

5 weeks	WKY	SHR	GJD-LD	GJD-MD	GJD-HD	CAP
1	281.6	295.7	262.1	308.4	311.4	313.7
2	268.3	312.9	265.3	309.3	318.2	340.2
3	296.6	296.2	305.3	292.6	319.3	304.7
4	305.4	287.5	338.6	331.6	292.4	308.3
5	291.9	312.6	309.5	305.3	317.8	306.9
6	333.8	292.6	292.4	279.4	294.6	296.3
7	279.7	290.6	340.5	329.3	301.7	332.1
Mean	293.90	298.30	301.96	307.99	307.91	314.60

6 weeks	WKY	SHR	GJD-LD	GJD-MD	GJD-HD	CAP
1	294.5	299.4	257.4	300.2	307.8	311.3
2	274.3	309.8	261.6	314.3	325.5	335.4
3	295.2	300.2	303.1	297.4	320.4	307.0
4	297.7	289.1	332.5	340.0	309.7	300.5
5	292.1	313.8	299.8	309.4	317.2	309.4
6	332.4	287.4	299.7	289.7	300.3	301.8
7	285.1	292.6	340.6	331.4	311.2	334.3
Mean	295.90	298.90	299.24	311.77	313.16	314.24

Endothelin-1	ng/L	WKY	SHR	GJD-LD	GJD-MD	GJD-HD	CAP
	1	14.92	147.91	84.22	72.50	73.37	67.05
	2	22.69	129.20	36.58	61.31	68.30	78.08
	3	30.58	153.23	107.67	95.98	81.89	76.21
	4	75.86	88.88	121.69	100.59	55.88	65.02
	5	53.86	167.76	86.33	73.54	69.16	86.61
	6	96.91	129.89	93.25	33.01	65.61	66.64
	7	62.25	84.00	43.45	62.99	52.43	88.81
	Mean	51.01	128.70	81.88	71.42	66.66	75.49

Angiotensin II	ng/L	WKY	SHR	GJD-LD	GJD-MD	GJD-HD	CAP
	1	242.55	688.98	397.62	460.63	386.65	525.90
	2	194.84	638.25	458.35	431.16	479.91	467.80
	3	210.10	585.63	632.97	480.86	312.37	319.83
	4	398.37	647.45	543.36	482.77	340.75	384.74
	5	328.52	564.58	510.01	617.13	452.95	400.22
	6	303.20	520.12	382.59	441.89	329.77	335.74
	7	205.20	775.04	457.42	322.48	276.49	322.59
	Mean	268.97	631.44	483.19	462.42	368.41	393.83

IL-1 beta	ng/L	WKY	SHR	GJD-LD	GJD-MD	GJD-HD	CAP
	1	25.12	71.80	25.51	36.87	28.73	34.48
	2	15.98	61.84	31.74	66.96	25.53	28.65
	3	28.56	52.29	50.83	54.22	31.27	35.81
	4	22.96	71.32	57.47	37.21	68.18	34.38
	5	36.70	55.30	54.51	65.30	36.41	38.25
	6	25.20	73.22	54.82	17.96	28.44	57.30
	7	16.59	68.30	51.63	27.64	35.11	25.52
	Mean	24.44	64.87	46.64	43.74	36.24	36.34

Interleukin- 6	ng/L	WKY	SHR	GJD-LD	GJD-MD	GJD-HD	CAP
	1	30.23	72.77	66.85	112.59	85.04	57.12
	2	73.30	99.13	73.89	37.65	48.24	86.35
	3	24.07	172.88	97.23	88.11	45.99	84.81
	4	39.74	119.36	122.12	49.32	82.10	57.11
	5	43.41	163.69	121.40	116.93	66.23	64.63
	6	52.81	78.67	67.52	104.53	55.13	73.54
	7	42.40	74.86	57.31	47.45	63.24	39.13
	Mean	43.71	111.62	86.62	79.51	63.71	66.10

TNF- alpha	ng/L	WKY	SHR	GJD-LD	GJD-MD	GJD-HD	CAP
	1	145.07	196.69	215.29	138.90	150.00	181.49
	2	93.66	267.56	103.80	152.34	167.51	238.14
	3	91.41	228.83	140.58	202.47	142.02	166.37
	4	115.47	225.83	145.47	117.19	190.97	118.55
	5	96.95	287.56	271.69	224.31	185.74	163.07
	6	124.51	210.15	153.35	135.72	129.27	138.25
	7	95.83	240.72	160.40	170.40	135.04	155.10
	Mean	108.99	236.76	170.08	163.05	157.22	165.85

Thoracic aorta Thickness(mm)	WKY	SHR	GJD-LD	GJD-MD	GJD-HD	CAP
1	0.11866	0.17535	0.15293	0.14168	0.12986	0.13146
2	0.12121	0.18909	0.14489	0.14239	0.12395	0.12387
3	0.11508	0.19033	0.14618	0.14294	0.13352	0.13445
4	0.11756	0.18035	0.15373	0.14378	0.12666	0.13056
5	0.12011	0.18849	0.14029	0.14449	0.12215	0.12297
6	0.11478	0.19163	0.14448	0.14004	0.13132	0.13375
7	0.11624	0.19099	0.15534	0.14113	0.13074	0.13132
Mean	0.11766	0.1866	0.14826	0.14235	0.12832	0.12977

IL-1 beta	WKY	SHR	GJD-LD	GJD-MD	GJD-HD	CAP
1	33413	58087	40113	44428	37019	40420
2	27941	57708	40655	38134	35310	40414
3	26091	66748	36593	41168	30390	38928
4	33335	52618	29616	44100	42068	36276
5	34957	65255	40372	42155	34513	34065
6	39644	63333	35284	32147	35915	35895
7	37560	65807	34941	31947	36041	27002
Mean	33277.3	61365.1	36796.3	39154.1	35893.7	36142.9

Interleukin- 6	WKY	SHR	GJD-LD	GJD-MD	GJD-HD	CAP
1	30721	44929	42586	62152	45546	45765
2	28120	46064	31922	55204	43237	39396
3	37848	49249	44219	46362	32340	38357
4	41583	44538	55408	51657	36439	36048
5	30231	64205	50661	46497	37780	45250
6	22282	55744	55089	45778	47436	39259
7	26213	58992	45605	44682	37339	32588
Mean	30999.7	51960.1	46498.6	50333.1	40016.7	39523.3

TNF- alpha	WKY	SHR	GJD-LD	GJD-MD	GJD-HD	CAP
1	5227	19516	10430	14804	14571	9394
2	1677	17712	8804	17129	9414	7810
3	5463	16333	10781	10297	4642	10748
4	4428	11381	18583	11966	7134	11602
5	4360	10868	13215	9089	4920	6087
6	7273	13365	9436	9490	4818	8507
7	6777	12262	13651	16866	7116	10335
Mean	5029.29	14491	12128.6	12805.9	7516.43	9211.86

Collagen- I	WKY	SHR	GJD-LD	GJD-MD	GJD-HD	CAP
1	1861	3918	2557	1302	1568	2617
2	715	5464	4144	4746	1238	2728
3	822	4022	1742	2071	1424	1077
4	699	2072	2389	2151	3078	1076
5	1056	2403	3433	1429	1147	1243
6	1266	2710	3077	1577	1876	1368
7	2441	3176	1157	2486	1820	1542
Mean	1265.71	3395	2642.71	2251.71	1735.86	1664.43

Collagen- III	WKY	SHR	GJD-LD	GJD-MD	GJD-HD	CAP
1	14515	26729	16495	26212	22626	18408
2	19040	31637	21817	18477	13083	20479
3	10761	25967	20917	15514	15116	19039
4	19039	23016	21086	15961	19060	16966
5	19607	26074	14159	18339	25319	20333
6	20151	23775	23508	16630	14686	17979
7	13667	18718	15644	16400	17927	20634
Mean	17185.5	26199.7	19663.7	18522.2	18315	18867.3

alpha- SMA	WKY	SHR	GJD-LD	GJD-MD	GJD-HD	CAP
1	8662	10752	12356	10136	8501	9554
2	9684	17527	12262	10936	10213	10167
3	6832	13165	10330	8608	9069	10153
4	7369	14253	10963	9299	9656	6319
5	9025	17909	11633	8439	8514	7158
6	8672	13392	10615	7735	7271	8784
7	6239	13081	10073	8936	8212	9464
Mean	8069	14297	11176	9155.57	8776.57	8799.86

IL-1 beta	WKY	SHR	GJD-LD	GJD-MD	GJD-HD	CAP
1	8406	19083	11036	5473	9766	8015
2	10244	17003	8340	9525	9056	6258
3	6641	16626	6194	8272	10318	9358
4	5998	17492	12776	9752	10060	11327
5	3263	22552	11451	5504	8393	10600
6	6270	21505	9151	10601	7651	6339
7	4151	24869	11028	9741	6272	10526
Mean	6424.65	19875.5	9996.62	8409.71	8788.08	8917.56

Interleukin- 6	WKY	SHR	GJD-LD	GJD-MD	GJD-HD	CAP
1	4315	5224	2212	3979	1795	1184
2	3309	2418	3464	3058	2473	1816
3	3373	4981	3116	3832	1763	897
4	359	5150	8002	2573	2961	3736
5	633	8211	3752	4441	2324	3066
6	390	9571	1511	1921	4002	2883
7	2477	9488	4150	3407	1125	2239
Mean	2122.43	6434.96	3744.06	3315.91	2348.99	2260.22

TNF- alpha	WKY	SHR	GJD-LD	GJD-MD	GJD-HD	CAP
1	13023	20831	10318	7695	10423	13245
2	9593	25618	14460	11668	13690	10660
3	6966	13916	17511	6415	10999	16275
4	6134	16320	10809	17762	9446	10687
5	5726	25207	12736	11438	12530	18804
6	13243	11727	12785	13705	13385	9623
7	7379	21393	10138	14579	9613	7262
Mean	8866.17	19287.6	12679.6	11894.6	11440.6	12365.2

Collagen- I	WKY	SHR	GJD-LD	GJD-MD	GJD-HD	CAP
1	7117	8238	7730	10063	6143	8739
2	3980	8258	5325	4027	5867	7785
3	4772	12629	6075	6512	7831	11340
4	9148	14782	6345	9625	4454	10419
5	5551	14410	9384	5815	7476	2351
6	3829	9373	6735	5011	8582	2489
7	3663	15091	6919	6469	4838	3848
Mean	5437.25	11825.8	6930.51	6788.86	6455.94	6710.06

Collagen- III	WKY	SHR	GJD-LD	GJD-MD	GJD-HD	CAP
1	20887	30118	16463	12562	7592	18922
2	6643	27111	14246	18112	16246	10798
3	20372	23575	12549	16540	10881	14467
4	5140	29637	16618	15690	7224	17974
5	20755	21268	17404	14229	20517	12961
6	10235	20251	18789	18197	15954	17644
7	8310	16035	23564	19482	20569	11058
Mean	13191.7	23999.1	17090.5	16401.7	14140.3	14832

alpha- SMA	WKY	SHR	GJD-LD	GJD-MD	GJD-HD	CAP
1	38336	76405	52895	41851	37674	56795
2	24207	64980	44576	46606	33776	40186
3	33586	67049	49867	49673	48314	54765
4	43879	58565	45878	42039	54000	47873
5	49221	75380	68250	51160	44179	63083
6	47831	61169	47731	53495	46971	61499
7	30281	77772	53007	48638	56337	62105
Mean	38191.7	68760	51743.4	47637.3	45893	55186.4

IL-1β	WKY	SHR	GJD-LD	GJD-MD	GJD-HD	CAP
1	1.00	2.37	1.45	1.38	1.10	1.34
2	1.00	2.21	1.87	1.62	1.26	1.69
3	1.00	2.80	1.83	1.89	1.65	1.81
Mean	1.00	2.46	1.72	1.63	1.34	1.61

Col-I	WKY	SHR	GJD-LD	GJD-MD	GJD-HD	CAP
1	1.00	6.61	4.21	3.11	2.66	3.09
2	1.00	6.35	3.19	2.86	2.68	4.71
3	1.00	5.14	2.73	2.60	1.47	1.62
Mean	1.00	6.03	3.38	2.86	2.27	3.14

TAK1	WKY	SHR	GJD-LD	GJD-MD	GJD-HD	CAP
1	1.00	4.53	3.12	2.37	1.76	1.77
2	1.00	3.54	2.42	2.37	1.81	1.93
3	1.00	5.30	3.38	2.31	1.64	1.61
Mean	1.00	4.46	2.97	2.35	1.74	1.77

IL-6	WKY	SHR	GJD-LD	GJD-MD	GJD-HD	CAP
1	1.00	2.32	1.35	1.26	1.05	1.41
2	1.00	3.43	2.29	1.46	1.17	1.77
3	1.00	2.31	2.03	1.70	1.02	1.18
Mean	1.00	2.69	1.89	1.47	1.08	1.45

Col-III	WKY	SHR	GJD-LD	GJD-MD	GJD-HD	CAP
1	1.00	3.24	2.05	1.76	1.31	1.82
2	1.00	2.88	2.56	1.97	1.83	1.57
3	1.00	4.14	3.13	2.15	1.94	1.81
Mean	1.00	3.42	2.58	1.96	1.69	1.73

IKB-α	WKY	SHR	GJD-LD	GJD-MD	GJD-HD	CAP
1	1.00	0.16	0.30	0.44	0.70	0.65
2	1.00	0.19	0.38	0.53	0.77	0.68
3	1.00	0.25	0.49	0.58	0.83	0.85
Mean	1.00	0.20	0.39	0.52	0.77	0.73

TNF-α	WKY	SHR	GJD-LD	GJD-MD	GJD-HD	CAP
1	1.00	2.74	1.36	1.28	1.17	1.11
2	1.00	3.08	1.93	1.85	1.51	1.81
3	1.00	4.09	3.23	2.98	1.48	1.41
Mean	1.00	3.31	2.18	2.04	1.39	1.44

α-SMA	WKY	SHR	GJD-LD	GJD-MD	GJD-HD	CAP
1	1.00	3.74	2.21	2.26	1.92	2.35
2	1.00	3.26	1.80	1.88	1.52	1.85
3	1.00	3.44	2.32	1.64	1.34	1.24
Mean	1.00	3.48	2.11	1.93	1.59	1.81

p65	WKY	SHR	GJD-LD	GJD-MD	GJD-HD	CAP
1	1.00	3.70	2.76	1.91	1.43	1.89
2	1.00	3.92	2.68	1.51	1.36	1.74
3	1.00	3.79	2.33	1.73	1.52	1.67
Mean	1.00	3.80	2.59	1.72	1.43	1.77

IL-1β	WKY	SHR	GJD-LD	GJD-MD	GJD-HD	CAP
1	1.00	4.09	1.74	1.71	1.30	1.52
2	1.00	4.64	2.16	1.50	1.06	1.22
3	1.00	2.32	2.29	1.41	1.44	1.33
Mean	1.00	3.68	2.06	1.54	1.27	1.36

Col-I	WKY	SHR	GJD-LD	GJD-MD	GJD-HD	CAP
1	1.00	3.84	2.37	1.87	1.53	1.60
2	1.00	4.60	2.03	2.04	1.77	1.65
3	1.00	3.27	1.77	2.00	1.82	1.78
Mean	1.00	3.90	2.06	1.97	1.71	1.68

TAK1	WKY	SHR	GJD-LD	GJD-MD	GJD-HD	CAP
1	1.00	3.06	2.24	2.02	1.78	1.72
2	1.00	4.06	3.10	2.16	1.75	1.83
3	1.00	4.29	2.41	1.72	1.39	1.43
Mean	1.00	3.80	2.58	1.97	1.64	1.66

IL-6	WKY	SHR	GJD-LD	GJD-MD	GJD-HD	CAP
1	1.00	3.16	1.57	1.34	1.36	1.43
2	1.00	4.64	2.78	1.95	1.60	1.42
3	1.00	2.63	2.39	1.50	1.13	1.09
Mean	1.00	3.47	2.25	1.60	1.36	1.31

Col-III	WKY	SHR	GJD-LD	GJD-MD	GJD-HD	CAP
1	1.00	2.16	1.68	1.67	1.34	1.33
2	1.00	2.81	2.31	2.06	1.63	1.80
3	1.00	3.00	1.96	1.99	1.77	1.78
Mean	1.00	2.66	1.99	1.91	1.58	1.64

IKB-α	WKY	SHR	GJD-LD	GJD-MD	GJD-HD	CAP
1	1.00	0.26	0.31	0.50	0.68	0.73
2	1.00	0.29	0.43	0.52	0.70	0.66
3	1.00	0.27	0.47	0.59	0.81	0.65
Mean	1.00	0.27	0.40	0.54	0.73	0.68

TNF-α	WKY	SHR	GJD-LD	GJD-MD	GJD-HD	CAP
1	1.00	2.82	2.62	2.48	1.98	1.70
2	1.00	3.04	2.16	1.66	1.47	1.56
3	1.00	3.00	2.34	1.79	1.28	1.66
Mean	1.00	2.96	2.37	1.98	1.58	1.64

α-SMA	WKY	SHR	GJD-LD	GJD-MD	GJD-HD	CAP
1	1.00	2.34	1.63	1.45	1.09	1.45
2	1.00	2.52	1.75	1.50	1.43	1.56
3	1.00	2.55	1.78	1.79	1.53	1.90
Mean	1.00	2.47	1.72	1.58	1.35	1.63

p65	WKY	SHR	GJD-LD	GJD-MD	GJD-HD	CAP
1	1.00	3.16	2.56	1.93	1.65	1.76
2	1.00	3.06	2.45	2.03	1.54	1.57
3	1.00	3.25	2.11	1.97	1.53	1.80
Mean	1.00	3.16	2.38	1.98	1.57	1.71

TAK1	WKY	SHR	GJD-LD	GJD-MD	GJD-HD	CAP
1	0.13214	0.99679	0.53621	0.36033	0.2582	0.27792
2	0.07813	0.60849	0.52202	0.41905	0.27321	0.27014
3	0.07195	0.71892	0.55067	0.35044	0.2214	0.2291
Mean	0.09407	0.77473	0.5363	0.37661	0.25094	0.25906

P65	WKY	SHR	GJD-LD	GJD-MD	GJD-HD	CAP
1	0.27645	1.29641	0.94451	0.83884	0.69089	0.68922
2	0.06884	1.03719	0.79232	0.48621	0.42357	0.42147
3	0.07233	0.90697	0.58418	0.42507	0.18554	0.18703
Mean	0.1392	1.08019	0.77367	0.58337	0.43333	0.43257

IKB- alpha	WKY	SHR	GJD-LD	GJD-MD	GJD-HD	CAP
1	1.00208	0.17246	0.28046	0.44219	0.67323	0.6941
2	1.06062	0.11662	0.16852	0.21964	0.42195	0.44269
3	0.8457	0.07187	0.12645	0.20401	0.39296	0.39445
Mean	0.96947	0.12032	0.19181	0.28861	0.49605	0.51041

TAK1	WKY	SHR	GJD-LD	GJD-MD	GJD-HD	CAP
1	0.08891	0.58291	0.43787	0.29306	0.17181	0.16042
2	0.12973	0.96037	0.64012	0.39541	0.22179	0.21477
3	0.13161	0.69889	0.47029	0.34315	0.23355	0.2476
Mean	0.11675	0.74739	0.51609	0.34388	0.20905	0.2076

P65	WKY	SHR	GJD-LD	GJD-MD	GJD-HD	CAP
1	0.12717	0.98544	0.82948	0.75743	0.39841	0.37612
2	0.2864	0.95156	0.75671	0.54188	0.4538	0.44445
3	0.23207	0.74242	0.5928	0.43986	0.32497	0.32154
Mean	0.21521	0.89314	0.72633	0.57973	0.39239	0.3807

IKB- alpha	WKY	SHR	GJD-LD	GJD-MD	GJD-HD	CAP
1	1.19362	0.18859	0.38359	0.64639	0.79263	0.811
2	0.99572	0.26914	0.42645	0.60038	0.75865	0.77323
3	1.20005	0.11449	0.22735	0.43717	0.69298	0.71098
Mean	1.1298	0.19074	0.3458	0.56131	0.74809	0.76507
