# Supplementary material for: GJD Modulates Cardiac/Vascular Inflammation and Decreases Blood Pressure in Hypertensive Rats
Source: Mediators Inflamm. 2022 Sep 17;2022:7345116. doi: 10.1155/2022/7345116 (PMC9509256; doi:10.1155/2022/7345116)

Heart Western Blot：

TAK1

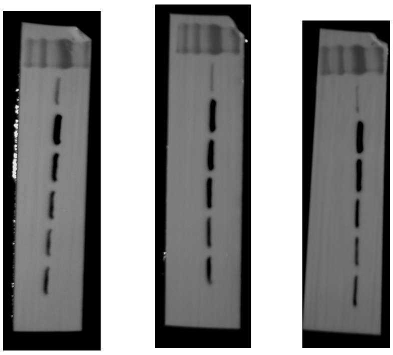

IKB- alpha

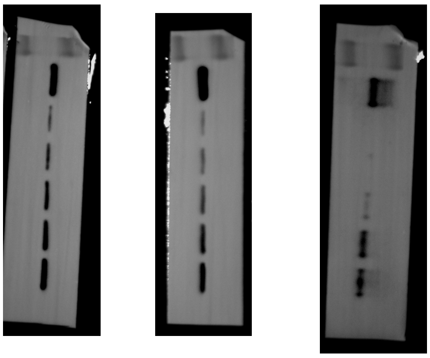

P65

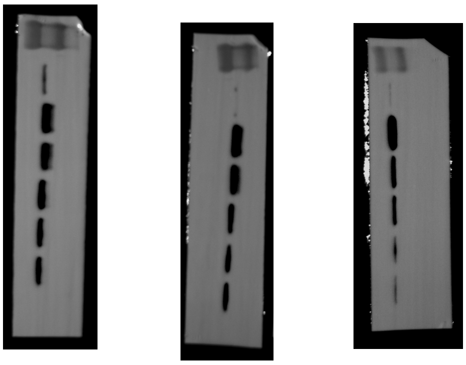

beta-actin

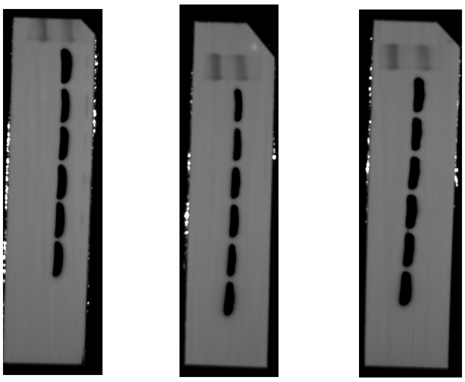

Thoracic aorta Western Blot：

TAK1

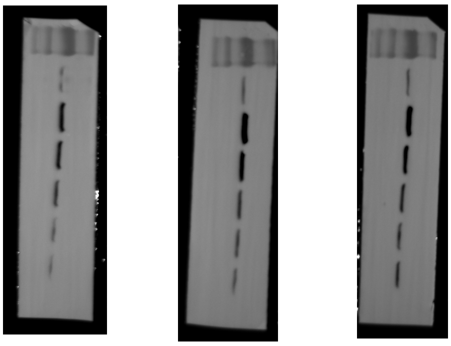

IKB- alpha

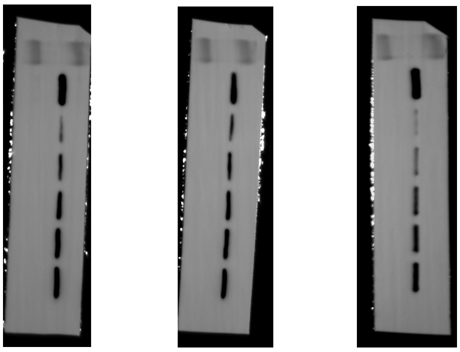

P65

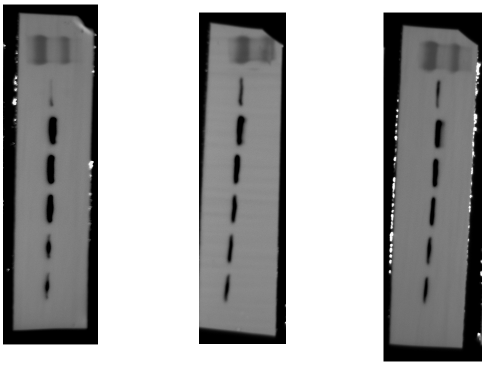

beta-actin

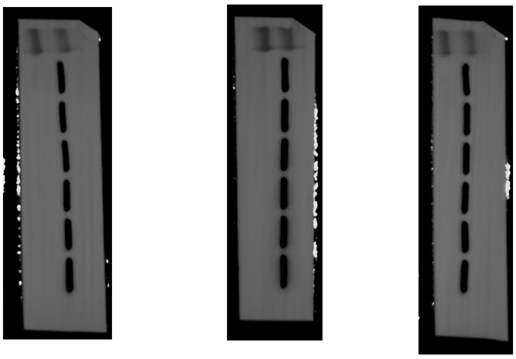

Supplement: Supplementary Materials — The supplementary materials which include all data used in this study in Supplementary Data 1 and western blot in Supplementary Data 2. [file 7345116.f1.zip › Supplementary Data 2.pdf]
